# Associations of combined lifestyle behaviors with all-cause and cardiovascular mortality in adults: A population-based cohort study in Jiangxi Province of China

**DOI:** 10.3389/fpubh.2022.942113

**Published:** 2022-10-28

**Authors:** Tao Wang, Congcong Ding, Wei Zhou, Lingjuan Zhu, Chao Yu, Xiao Huang, Huihui Bao, Xiaoshu Cheng

**Affiliations:** ^1^Department of Cardiovascular Medicine, The Second Affiliated Hospital of Nanchang University, Nanchang, China; ^2^Center for Prevention and Treatment of Cardiovascular Diseases, The Second Affiliated Hospital of Nanchang University, Nanchang, China; ^3^Jiangxi Provincial Cardiovascular Disease Clinical Medical Research Center, Nanchang, China

**Keywords:** lifestyle behaviors, all-cause mortality, cardiovascular mortality, cohort study, Jiangxi Province

## Abstract

**Background:**

Data are limited on the impact of combined lifestyle behaviors on mortality in Jiangxi Province, China.

**Objective:**

The study examined the association between combined lifestyle behaviors and all-cause and cardiovascular disease (CVD) mortality in Jiangxi province.

**Methods:**

The baseline survey was completed in Jiangxi Province from November 2013 to August 2014. We conducted a follow-up on 12,608 participants of 35 years of age or older from July 2019 to October 2020. Four known lifestyle behaviors were evaluated: alcohol consumption, smoking, diet (AHEI scores), and physical activity. Cox regression analysis was performed to determine the association of combined lifestyle behaviors with all-cause and CVD mortality.

**Results:**

During 65,083 person-years of follow-up, among the 11,622 participants (mean age 59.1 years; 40.1% men) 794 deaths occurred, including 375 deaths from CVD disease in this study. Compared to the favorable lifestyle group, the adjusted HR of all-cause mortality was 1.25 (95% CI, 1.03–1.53) for the intermediate lifestyle group and 1.37 (95% CI, 1.11–1.71) for the unfavorable lifestyle group. Compared to the favorable lifestyle group, the adjusted HR of CVD mortality was 1.50 (95% CI, 1.11–2.03) for the intermediate lifestyle group and 1.58 (95% CI, 1.14–2.20) for the unfavorable lifestyle group. Significant interactions of lifestyle and BMI (*P* for interaction <0.05) with the risk of all-cause mortality and CVD mortality were observed.

**Conclusion:**

In the current study, we reaffirm the associations of combined lifestyle factors with total and CVD mortality in Jiangxi Province, our data suggest that an unfavorable lifestyle was associated with a substantially increased risk of all-cause and CVD mortality.

## Introduction

The four health behaviors mentioned in Life's Simple 7 scores recommended by the American Heart Association (1), or the concept of healthy lifestyle medicine—a timeless medicine (2), both indicate that we should pay attention to lifestyle behaviors. Meanwhile, a large proportion of global deaths from all causes and diseases has been attributed to modifiable lifestyle behaviors. Moreover, studies also demonstrated considerably lower risks of cardiovascular and other mortality ascribed to a healthy lifestyle (3–6). Healthy lifestyle behaviors include the adoption of a healthy diet, cessation of smoking, moderate alcohol intake, and regular physical activity (4). Early research revealed that tobacco use, diet, physical activity patterns, and alcohol were the actual leading causes of death, accounting for ~80% of premature deaths (7). And there are more and more epidemiological studies that have shown that healthy behaviors are associated with a lower risk of premature death from all-causes and cardiovascular disease (CVD). However, the majority of the studies were conducted in western populations and areas with high economic and medical levels in China (8–10). There has been limited evidence on the impact of combined lifestyle behaviors on mortality in Jiangxi Province of China. In China, there are large differences in the dietary habits and lifestyles of residents in different provinces. For example, the southern population is low in smoking (11) and dietary sodium intake (12, 13), and high in energy intake (14). Therefore, it is necessary to study the associations of combined lifestyle behaviors with the risks of all-cause and CVD mortality in Jiangxi Province, whose lifestyle patterns are different from other provinces in China.

Lifestyle factors are often interrelated and associated with multiple non-communicable diseases, and investigating the combined effects of multiple lifestyle factors, which could reflect the benefits of overall healthy lifestyles, might be more appropriate to account for interactions between lifestyle factors. Therefore, evaluating the associations of combined lifestyle factors with total and CVD mortality could help governments and other organizations to formulate policies and guidelines tailored to local people's preferences, so as to facilitate their adopting healthy lifestyles.

This study aimed to examine the associations of combined lifestyle behaviors with the risks of all-cause and CVD mortality among adults in Jiangxi Province.

## Methods

### Study setting and population

**Figure 1:**
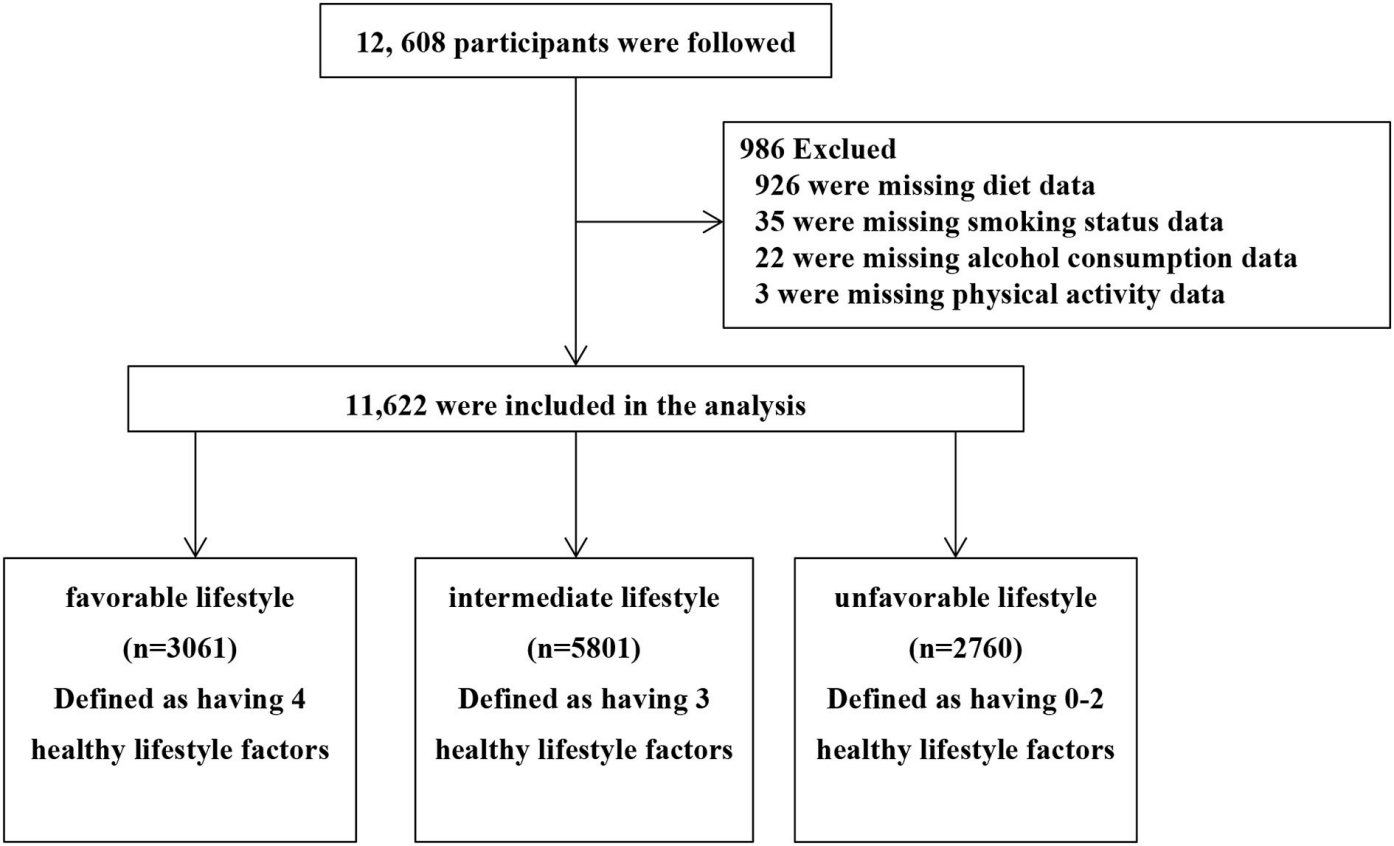
Study flow diagram.

Previous studies (15, 16) have described the population and methods (Project No. 2011BAI11B01). From November 2013 to August 2014, a multistage stratified random sampling (SRS) method was used to select eight counties/districts in Jiangxi Province. Using the same SRS method, a given number of participants from each of the 14 gender/age strata (male/female and aged 15–24, 25–34, 35–44, 45–54, 55–64, 65–74, ≥75) were selected from communities or villages using the lists compiled from the local government registers of households (17). Baseline data collected at enrollment include patient demographic characteristics, personal medical history, and data, while lifestyle information (such as smoking, alcohol consumption, diet, and physical activity status) was collected directly from patients using a questionnaire. Briefly, this study conducted a follow-up for people 35 years of age or older in rural and urban areas of Jiangxi Province. The content of the follow-up included survival, cause of death, and time of death. There are three follow-up methods. First, the survival status of the participants was determined by Jiangxi Provincial Center for Disease Control; second, follow-up by telephone Q&A; third, check the list of records at each township central hospital. As a consequence, a total of 12,608 participants completed follow-up from July 2019 to October 2020. Missing alcohol consumption data (*n* = 22), smoking status data (*n* = 35), as well as those who did not complete the diet questionnaire (*n* = 926), and physical activity questionnaire (*n* = 3) were excluded. Finally, a total of 11,622 participants were analyzed (Figure 1). All participants who provided written informed consent were enrolled in the study. If the participants were unable to write, fingerprinting would be used. This survey was approved by the Medical Research Ethics Committee of the Second Affiliated Hospital of Nanchang University and the Fuwai Cardiovascular Hospital (Beijing, China).

### Data collection

To ensure standardization and high quality of epidemiological information data, a comprehensive operational manual with a set of training slides was developed. All research investigators have completed a training program that oriented them both to the aims of the study and the specific tools and methodologies employed. Participants were required to complete a standardized questionnaire that was developed by the national coordinating center of the Fuwai Hospital (Beijing, China) through face-to-face interviews with trained staff.

### Assessment of lifestyle behaviors

We included four lifestyle behaviors: smoking, alcohol intake (18), diet quality, and physical activity. A score in the top 40% of the Alternative Healthy Eating Index-2010 (AHEI-2010) score (19) (Supplementary Table 1, non-current smoking, never or light drinking, and regular physical activity were considered as healthier lifestyle factors (Supplementary Table 2). As we decided a priori to include alcohol as a separate factor, it is not included in the AHEI-2010 score in this analysis. Similar to previous research (20), we divided lifestyles into three categories: favorable (four healthy lifestyle factors), moderate (three healthy lifestyle factors), and unfavorable (0–2 healthy lifestyle factors). Given that body mass index (BMI) was not considered a behavior but rather an intermediate health outcome influenced by several lifestyle behaviors (21) and that BMI may no longer serve as a valid measure of obesity, it was not included in the lifestyle score to minimize reverse causation bias (22).

### Ascertainment of deaths

All eight study areas are covered by the Jiangxi Province Disease Surveillance Points system, which provides cause-specific mortality data. Moreover, links with DSP death registries and local residential records, combined with home phone answering and contact with township hospital follow-up, were used to ascertain participants' vital status. Primary endpoints for current analyses included all-cause and CVD mortality resulting from either heart disease or cerebrovascular disease (ICD-10 codes I00-09, 11, 13, 20-25, 60-69). The follow-up for death in this cohort was at least 98% complete. A physician reviewed death certificates or medical records to classify the cause of death according to ICD-10 in the study.

### Statistical analysis

Baseline characteristics of the study population were presented stratified by lifestyle categories. Data are expressed as mean ± standard deviation (SD) for continuous variables and frequency (%) for categorical variables. Comparisons among different lifestyle category groups were performed using a one-way ANOVA test (continuous variables) or the chi-square test (categorical variables) accordingly. Lifestyle categories were analyzed as a categorical variable with favorable lifestyle being the reference group. Multiple Cox proportional hazards regression analysis was used to analyze the relationship between lifestyle and all-cause mortality, and the results were presented as hazard ratio (HR) and 95% confidence interval (CI). P for trend was calculated by assigning the categorical variables of favorable, intermediate, and unfavorable lifestyles as 0, 1, and 2, and then converting them into continuous variables. The adjusted variables selection is based on the importance of the impact on the clinical outcome, the existence of statistical significance in the univariate analysis, and on the basis of their associations with the outcomes of interest or a change in effect estimate of more than 10% (23). Cumulative mortality was estimated by the Kaplan-Meier method, and any differences in cumulative mortality were evaluated with log-rank tests. Finally, stratified analyses were conducted, with stratification by age, gender, BMI, waist circumference (WC), hypertension, and stroke, to find out potential subgroups in which there was a significant association between lifestyle and all-cause and CVD mortality. To demonstrate the robustness of our findings, we excluded deaths that occurred within 1 year of follow-up duration to examine whether our analyses were impacted by reverse causation bias. In addition, we did the analysis excluding participants with stroke and myocardial infarction at baseline.

All data analyses were performed using statistical packages R and (http://www.R-project.org, The R Foundation) and Empower (R) (www.empowerstats.com; X&Y Solutions, Inc., Boston, MA). A two-sided *P*-value of <0.05 was considered to be statistically significant.

## Results

Among the 11,622 participants, 35–97 years of age (mean age 59.1 years; 40.1% men), in this study, 3,061 (26.3%) people were categorized as favorable lifestyle group, 5,801 (49.9%) people were categorized as intermediate lifestyle group, and 2,760 (23.7%) people were categorized as unfavorable lifestyle group. During 65,083 person-years of follow-up, 794 deaths occurred including 375 deaths from CVD. The characteristics of the study participants in lifestyle categories are shown in Table 1. At baseline, the unfavorable lifestyle groups were more likely to be men, older, fatter waist, higher rural occupancy rate; were more likely to have hypertension and stroke; and less likely to take anti-hypertensive drugs, compared with those with a higher rural occupancy rate.

**Table 1:** Baseline characteristics of all participants stratified by categories of lifestyle factors.

**Characteristics**	**Total (*n =* 11,622)**	**Lifestyle categories**			***P*-value**
		**Favorable lifestyle (*n =* 3061)**	**Intermediate lifestyle (*n =* 5801)**	**Unfavorable lifestyle (*n =* 2760)**	
Age (years), mean (SD)	59.1 (13.3)	57.5 (12.9)	59.2 (13.3)	60.7 (13.5)	<0.001
Male, *n* (%)	4,664 (40.1)	698 (22.8)	1,884 (32.5)	2,082 (75.4)	<0.001
Rural, *n* (%)	5,675 (48.8)	1,383 (45.2)	2,907 (50.1)	1,385 (50.2)	<0.001
**Employment status**, ***n*** **(%)**					<0.001
Working	3,869 (33.6)	1,097 (36.1)	1,809 (31.6)	963 (35.2)	
Retired	1,784 (15.5)	629 (20.7)	862 (15.0)	293 (10.7)	
Other	5,845 (50.9)	1,305 (43.0)	3,048 (53.2)	1,474 (53.9)	
BMI(kg/m^2^), mean (SD)	23.2 (3.7)	23.3 (3.5)	23.2 (3.6)	22.9 (3.9)	<0.001
Waist circumference (cm), mean (SD)	80.0 (9.4)	79.5 (9.0)	79.9 (9.3)	80.9 (9.7)	<0.001
Hypertension, *n* (%)	3,999 (34.4)	999 (32.6)	1,983 (34.2)	1,017 (36.8)	0.003
Anti-hypertensive drugs, *n* (%)	1,076 (7.3)	32.1 (10.5)	502 (8.7)	253 (9.2)	0.017
Self-reported stroke, *n* (%)	212 (1.8)	43 (1.4)	100 (1.7)	69 (2.5)	0.005
**Healthy lifestyle factors**, ***n*** **(%)**					
Non-current smoking	9,354 (80.5)	3,061 (100.0)	5,219 (90.0)	1,074 (38.9)	<0.001
Never or light drinking	10,642 (91.6)	3,061 (100.0)	5,631 (97.1)	1,950 (70.7)	<0.001
Regular physical activity	9,821 (84.5)	3,061 (100.0)	5,322 (91.7)	1,438 (52.1)	<0.001
Healthy diet	4,664 (40.1)	3,061 (100.0)	1,231 (21.2)	372 (13.5)	<0.001

BMI, Body Mass Index; SD, Standard deviation.

Table 2 shows the HR and 95% CI values of the relationship between lifestyle and all-cause and CVD mortality in the crude model, model 1 (partially adjusted), and model 2 (fully adjusted). Compared to the favorable lifestyle group, the unfavorable lifestyle groups had a 44% higher risk of all-cause mortality (HR: 1.44; 95% CI: 1.17–1.78) and a 65% higher risk of cardiovascular mortality (HR: 1.65; 95% CI: 1.19–2.27) after adjusting for age and sex. In model 2, the multivariable-adjusted HRs for all-cause mortality and CVD mortality for the unfavorable lifestyle groups were 1.37 (95% CI: 1.11–1.71) and 1.58 (95% CI: 1.14–2.20), respectively. In addition, we also presented the association of each lifestyle factor with all-cause and CVD mortality in Supplementary Table 3. The results remained similar when deaths that occurred within 1 year of follow-up duration were excluded (Supplementary Table 4), or participants with stroke and myocardial infarction were excluded at baseline (Supplementary Table 5).

**Table 2:** Risk of incident all-cause and cardiovascular disease mortality according to lifestyle categories.

**Lifestyle categories**	**Cases/controls**	**Crude model**		**Model 1**		**Model 2**	
		**HR (95%CI)**	***P*-value**	**HR (95%CI)**	***P*-value**	**HR (95%CI)**	***P*-value**
**All-cause mortality**							
favorable lifestyle	141/2,920	Ref.		Ref.		Ref.	
Intermediate lifestyle	384/5,417	1.50 (1.24,1.82)	<0.001	1.27 (1.05, 1.55)	0.014	1.25 (1.03, 1.53)	0.024
unfavorable lifestyle	269/2,491	2.25 (1.84,2.76)	<0.001	1.44 (1.17, 1.78)	0.001	1.37 (1.11, 1.71)	0.004
P for trend		<0.001		0.001		0.005	
**CVD mortality**							
favorable lifestyle	59/3,002	Ref.		Ref.		Ref.	
Intermediate lifestyle	190/5,611	1.77 (1.32, 2.37)	<0.001	1.50 (1.12, 2.01)	0.006	1.50 (1.11, 2.03)	0.008
unfavorable lifestyle	126/2,634	2.52 (1.85, 3.43)	<0.001	1.65 (1.19, 2.27)	0.002	1.58 (1.14, 2.20)	0.006
P for trend		<0.001		0.004		0.011	

Model I: adjusted for age and gender.

Model II: adjusted for age, gender, BMI, waist circumference, hypertension, anti-hypertensive drugs, and stroke.

**Figure 2:**
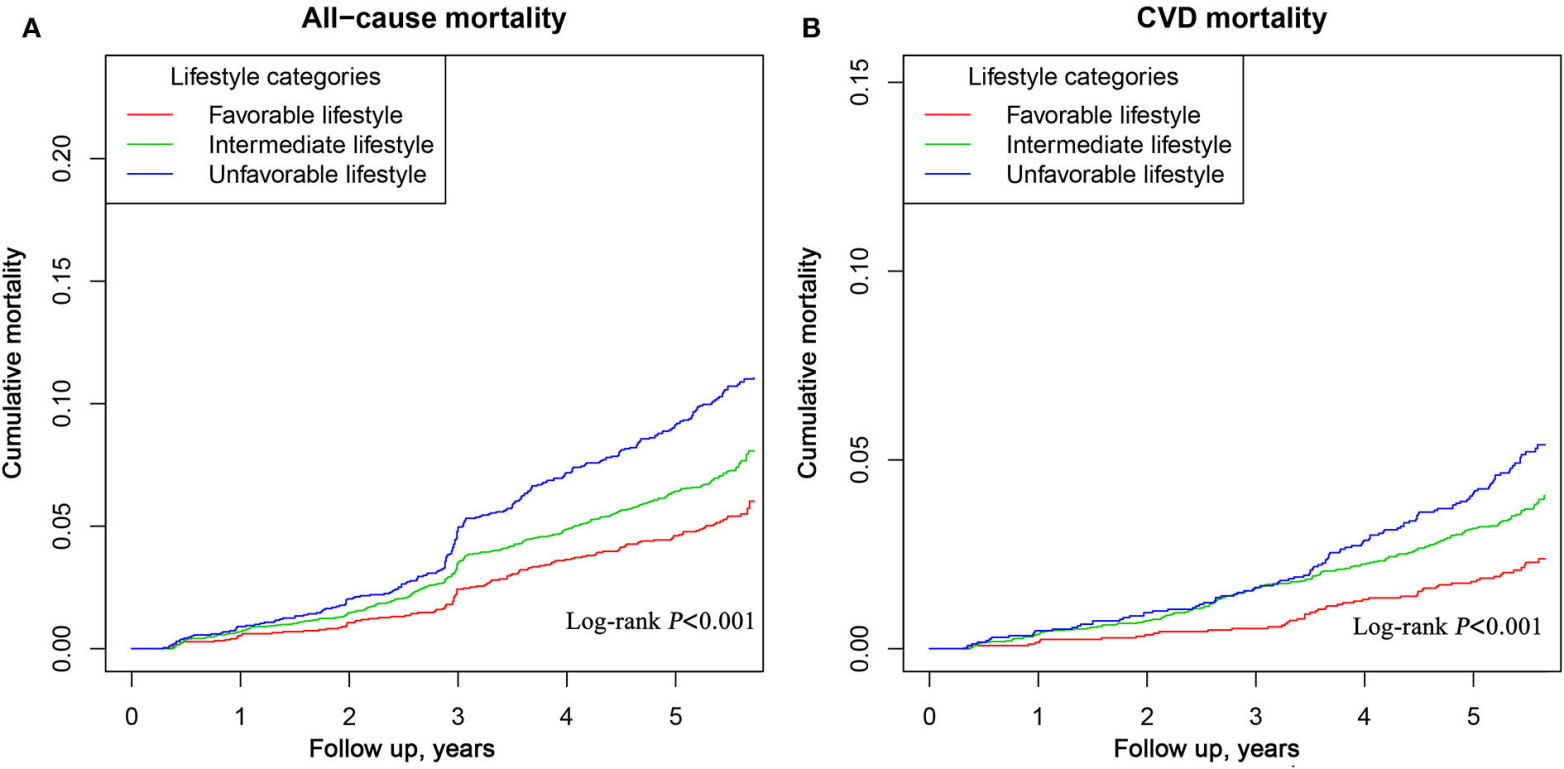
Kaplan-Meier curves of all-cause **(A)** and CVD **(B)** mortality among participants in Jiangxi Province, China.

**Figure 3:**
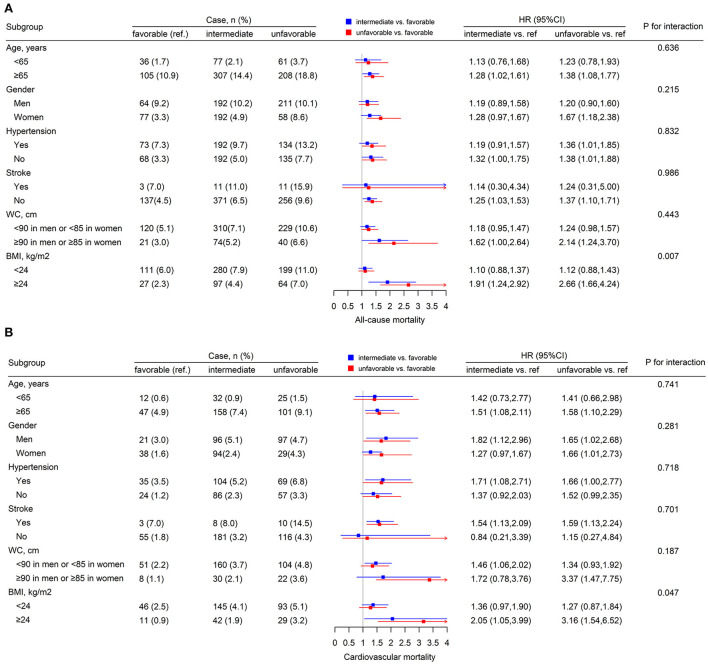
The subgroup analysis for the lifestyle on all-cause **(A)** and CVD **(B)** mortality. Each subgroup analysis adjusted, if not stratified, for age, gender, BMI, waist circumference, hypertension, stroke, and anti-hypertensive drugs.

Figure 2 shows the Kaplan-Meier curves of the cumulative all-cause (Figure 2A) and CVD (Figure 2B) mortality stratified by lifestyle categories. All-cause and CVD mortality between each of the three lifestyle groups was significantly different (log-rank test, *P* < 0.001). As shown in Figure 3, stratified analyses were conducted with stratification by age, gender, BMI, WC, hypertension, and stroke. There were no significant interactions of lifestyle and age, gender, WC, hypertension, and stroke on the risk of all-cause mortality. Significant interactions of lifestyle and BMI (*P* for interaction <0.05) with the risk of all-cause and CVD mortality were observed. In addition, The results remained similar when deaths that occurred within 1 year of follow-up duration were excluded (Supplementary Figure 1).

## Discussion

The current study evaluates the association between combined lifestyle behaviors and all-cause and CVD mortality among adults in Jiangxi Province and six different population subgroups. We reaffirm the importance of healthier lifestyle behaviors. In 11,622 men and women, more unhealthy lifestyle behaviors were associated with a considerably higher risk of death. Compared to the favorable lifestyle group, the intermediate and unfavorable lifestyle group experienced an increased risk of 1.25 and 1.37 (*P* for trend = 0.005) for all-cause mortality and 1.50 and 1.58 (*P* for trend = 0.011) for CVD mortality, respectively. Compared with the intermediate lifestyle and unfavorable lifestyle groups, favorable lifestyle group patients had a significantly higher survival probability. Moreover, similar results can be observed in participants with a history of hypertension and stroke. And another finding in this research is that there is an interaction between lifestyle and death in the subgroup of BMI, which is consistent with the conclusion mentioned in a previous article (24).

Our results are consistent with previous studies. A recently published meta-analysis study (25) of combined lifestyle factors and all-cause mortality and cardiovascular disease showed that compared with the participants with the least healthy lifestyles, those with the healthiest lifestyles had lower risks of all-cause mortality (HR = 0.45, 95% CI 0.41–0.48, 74 studies with 2,584,766 participants), cardiovascular mortality (HR = 0.42, 95% CI 0.37–0.46, 41 studies with 1,743,530 participants). The associations were primarily significant and consistent among individuals from different continents, racial groups, and socioeconomic backgrounds.

Although people commonly recognize the importance of lifestyle factors in preventing death, there is limited research on this topic in the Jiangxi population based on population-based cohort analysis. We did identify eight reports from the Chinese population. The earlier Shanghai Women's Health Study (8) showed that recruited 71,243 women (40–70 years of age, mean follow-up = 9.00 years) and observed that compared to women with a score of zero (least healthy), HRs (95% CI) for women with 4–5 healthy lifestyle factors were 0.57 (0.44–0.74) for total mortality and 0.29 (0.16–0.54) for CVD mortality. After that, Zhang et al. (9) used data from the Shanghai Men's Health Study, which included 61,480 men (40–74 years of age, mean follow-up = 9.29 years), and found that the HRs of men with four risk practices (including smoking, heavy alcohol use, unhealthy diet, and physical inactivity) comparing to those with zero were 2.92 (95%CI: 2.53, 3.38) for all-cause mortality and 3.15 (95%CI: 2.44, 4.05) for CVD mortality. Lin et al. (26) included 5,686 patients with type 2 diabetes (mean follow-up = 4.0 years) and found patients with three or more points (lifestyle behaviors consisting of smoking, alcohol drinking, physical inactivity, and carbohydrate intake) were at a 3.50-fold greater risk of all-cause mortality (95% CI: 2.06–5.96) and 4.24-fold (95% CI: 1.20–14.95) of CVD-specific mortality; similar results were seen in the Taichung Diabetes Study. Furthermore, Zhu et al. (27) examined the association between lifestyle risk factors and mortality in a China Kadoorie Biobank study (~0.5 million adults aged 30–79 years), after 10.2 years of follow-up, and found that compared with participants without any healthy factors, the HRs of participants with five healthy factors were 0.32 (95% CI: 0.28, 0.37) for all-cause mortality and 0.42 (95% CI: 0.26, 0.67) for CVD mortality. The Singapore Chinese study (28) shows that adopting five healthy (including a healthy diet, nonsmoking status, light to moderate alcohol drinking, being physically active, and having optimal BMI) vs. none was associated with a lower risk of all-cause and cause-specific mortality, and the HRs (95% CI) was 0.38 (0.29, 0.51) for all-cause mortality and 0.26 (0.13, 0.52) for CVD mortality. At the same time, Wu et al. (10) recruited participants from the Yinzhou Health Information System in Ningbo, Zhejiang Province and shows that adopting four healthy (including a standard BMI, never smoking, never drinking, and physical activity) vs. none was associated with a higher risk of all-cause and cause-specific mortality, and the HRs (95% CI) was 1.87 (1.77, 1.98) for all-cause mortality, 1.51 (1.35, 1.68) for CVD mortality. Besides, Jin S et al. (29) examined the association between lifestyle risk factors and mortality in the substudy of the Chinese Longitudinal Healthy Longevity Survey (2,039 older adults ≥65 years), and found that compared to participants without any healthy lifestyle factors, those with 5 healthy lifestyle factors had an 85% lower risk of mortality (HR = 0.15, 95% CI: 0.04, 0.60). The latest results of the Dongfeng-Tongji cohort, Lu et al. (13) included 14,392 patients with hypertension (mean follow-up = 7.3 years) and found improvement in lifestyle score after hypertension diagnosis was associated with lower risk of all-cause mortality (HR, 0.52; 95% CI, 0.36–0.76) and CVD mortality (HR, 0.53; 95% CI, 0.30–0.94). Overall, the risk of total and cause-specific mortality increased with the increment of risk score.

Although the HRs obtained by our research are inconsistent with the above-mentioned research risk values (increasing the risk of all-cause death by 43–394% and CVD death by 58–324%), these inconsistent results can be attributed to differences in the study population, the definition of the healthy lifestyle, and duration of follow-up. Importantly, as described by our research results, there is a large amount of evidence that the trend toward promoting a healthy lifestyle and reducing the risk ratio of death is very consistent. This consistency provides a strong basis for policy and public health practice. Modest, sustained improvements to lifestyle behaviors that could have a considerable impact at both the individual and population level were important public health priorities. Therefore, these health behaviors must be given priority to effectively reduce the economic burden of China's medical treatment.

There is still little understanding of the potential biological mechanisms in the causal relationship between lifestyle factors and mortality. For example, Nicola Veronese et al. (24) mentioned a series of metabolic and molecular alterations induced by exercise training, healthier diets rich in vitamins and phytochemicals, and avoidance of smoking. Bonaccio et al. (30) examined the potential contribution of traditional, inflammatory, and novel markers of CVD risk in explaining the associations of healthy lifestyles with mortality rates. However, considering the large population in Jiangxi, if we do not take active actions to solve this public health problem, more and more Jiangxi people with undesirable lifestyle behaviors and risk factors will considerably increase the public health burden. So, this topic should be explored more extensively in future studies.

### Strengths and limitations

There were several advantages to the current study. First, we additionally analyzed the impact of combined lifestyle behaviors in specific high-risk populations (e.g., elderly, hypertension, and stroke) on risks of all-cause and CVD mortality. Second, the results observed in our study also contain information about clinical treatments like anti-hypertension drugs. The answer to this question is particularly important because many people in the world suffer from diseases and take medication. Such results may indicate that no matter before or after the disease, improving lifestyle has a significant impact on lifelong health. And it can further illustrate the universal importance of improving lifestyle among different groups and different regions of the population including Jiangxi Province.

Several limitations should be acknowledged. First, lifestyle behaviors were self-reported and were subject to measurement error and desirability bias. Second, the present analyses only used information on lifestyle factors collected at baseline and could not necessarily account for the impact of long-term lifestyle patterns. Third, although we carefully adjusted for potential confounders, we cannot entirely rule out the possibility of residual confounding by unmeasured factors, such as diabetes and lipid abnormality. Fourth, we used a simple dichotomies algorithm to combine the lifestyle factors into a composite, which might be problematic since different lifestyle factors may carry differential weights for such a combination. Fifth, our estimates may not be readily generalized to other populations or Chinese populations living in other areas because of different distributions of lifestyle factors and population structure and characteristics. Last, we had a relatively short follow-up period (mean of 5.6 years) and a limited number of deaths.

## Conclusion

In the current study, we reaffirm the associations of combined lifestyle factors with total and CVD mortality in Jiangxi Province and our data suggest that an unfavorable lifestyle was associated with substantially increased risk of all-cause and CVD mortality. Given the great disease burden of chronic disease in Jiangxi Province, governments and health organizations should give high priority to the promotion of healthy lifestyles.

## Strengths and limitations of this study

Strengths: Our data suggest that an unfavorable lifestyle was associated with a substantially increased risk of all-cause and CVD mortality in the Jiangxi Province population, whose lifestyle patterns are different from other provinces in China.

The subgroup additionally analyzed the impact of combined lifestyle behaviors in specific high-risk populations (e.g., elderly, hypertension, and stroke) on all-cause and CVD mortality risks in the Jiangxi Province population.

This study took into account a clinically relevant potential confounding variable: anti-hypertension drugs.

Limitations: The lifestyle behaviors were self-reported which has the possibility to lead to measurement error and desirability bias.

This study only used information on lifestyle factors collected at baseline and could not necessarily account for the impact of long-term lifestyle patterns.

## Data availability statement

The datasets generated and analyzed during the current study are not publicly available due to the the Second Affiliated Hospital of Nanchang University regulations, but are available from the corresponding author on reasonable request. Requests to access the datasets should be directed to XC Email: xiaoshumenfan126@163.com, Fax: 0086-0791-86262262.

## Ethics statement

This study was approved by the Medical Research Ethics Committee of the Second Affiliated Hospital of Nanchang University and the Fuwai Cardiovascular Hospital (Beijing, China) before the start of the work (Ethics Number 2012402). The patients/participants provided their written informed consent to participate in this study.

## Author contributions

XC obtained funding. XC and HB conceived the study, participated in the design, and revised the manuscript. TW, CD, WZ, LZ, CY, and XH participated in the design, collected the data, performed statistical analyses, and drafted the manuscript. CD and WZ conducted the analysis. All authors read and approved the final manuscript.

## Funding

This work was supported by the Jiangxi Science and Technology Innovation Platform Project (20165BCD41005), the Jiangxi Provincial Natural Science Foundation (20212ACB206019), the Science and Technology Innovation Base Construction Project (20221ZDG02010), the Key R&D Projects, Jiangxi (20203BBGL73173), the Jiangxi Provincial Health Commission Science and Technology Project (202130440, 202210495, and 202310528), the Jiangxi Provincial Drug Administration Science and Technology Project (2022JS41), and the Fund project of the Second Affiliated Hospital of Nanchang University (2016YNQN12034, 2019YNLZ12010, 2021efyA01, and 2021YNFY2024).

## Conflict of interest

The authors declare that the research was conducted in the absence of any commercial or financial relationships that could be construed as a potential conflict of interest.

## Publisher's note

All claims expressed in this article are solely those of the authors and do not necessarily represent those of their affiliated organizations, or those of the publisher, the editors and the reviewers. Any product that may be evaluated in this article, or claim that may be made by its manufacturer, is not guaranteed or endorsed by the publisher.
