# Supplementary material for: Associations of combined lifestyle behaviors with all-cause and cardiovascular mortality in adults: A population-based cohort study in Jiangxi Province of China
Source: Front Public Health. 2022 Oct 28;10:942113. doi: 10.3389/fpubh.2022.942113 (PMC9651958; doi:10.3389/fpubh.2022.942113)
Supplement: Supplementary file 1 [file Data_Sheet_1.PDF]

Supplement table 1. The AHEI-2010 scoring method and mean scores[1]

Component	Criteria for minimum score (0)	Criteria for maximum score (10)
Vegetables[2], servings/d	0	≥5
Fruit[3], servings/d	0	≥4
Whole grains[4], g/d	0	
Women		75
Men		90
Sugar-sweetened beverages and fruit juice[5], servings/d	≥1	0
Nuts and legumes[6], servings/d	0	≥1
Red/processed meat[7], servings/d	≥1.5	0
trans Fat, % of energy	≥4	≤0.5
Long-chain (n-3) fats (EPA + DHA)[8], mg/d	0	250
PUFA[9],% of energy	≤2	≥10
Sodium[10], mg/d	Highest decile	Lowest decile
Alcohol[11], drinks/d		
Women	≥2.5	0.5-1.5
Men	≥3.5	0.5-2.0
Total	0	110

[1]AHEI, Alternate Healthy Eating Index

[2]We considered 5 servings/d as ideal, which reflects the upper range of current dietary guidelines and is consistent with intervention studies of intermediate CVD risk factors. One serving is 0.5 cup of vegetables or 1 cup of green leafy vegetables (1 cup = 236.59 g).

[3]We considered 4 servings/d to be ideal. One serving is 1 medium piece of fruit or 0.5 cup of berries (1 cup = 236.59 g)

[4]We used grams of whole grains, which accounts for the variability of the percentages of whole grain in various "whole grain" products. One serving of a 100% whole-grain product (i.e., 0.5 cup of oatmeal or brown rice) contains; 15 – 20 g of whole grains (per dry weight). We considered 75 g/d to be optimal (5 servings/d) for women and 90 g/d (6 servings/d) to be optimal for men on the basis of current guidelines for total grains.

[5]Intake of sugar-sweetened beverages, including soda and fruit drinks. We considered ≥1 serving/d to be the least optimal on the basis of the associations in the literature. One serving is 8 oz (1 oz = 28.35 g).

[6]Nuts, legumes, and vegetable protein (e.g., tofu) are important sources of protein and contain important constituents such as unsaturated fat, fiber, copper, magnesium, plant sterols, and other nutrients. We considered 1 serving/d to be ideal on the basis of the AHEI recommendations and the current literature. One serving is 1 oz (1 oz = 28.35 g) of nuts or 1 tablespoon (15 mL) of peanut butter.

[7]Less than 1 serving/mo was considered to be ideal, with an upper limit of ≥1.5 servings/d. One serving is 4 oz of unprocessed meat or 1.5 oz of processed meat (1 oz = 28.35 g).

[8]Because of the strength and consistency of fish and EPA + DHA on cardiac arrhythmias and CVD, we included this nutrient in the AHEI-2010 score. The cutoff for optimal intake (250 mg/d) is ;2 4-oz servings of fish /wk, which is consistent with current guidelines (1 oz = 28.35 g).

[9]We gave the highest score to individuals with ≥10% of total energy intake from PUFA on the basis of current guidelines from the USDA and the AHA. PUFA does not include EPA or DHA intake. [10]The cutoffs for sodium were based on deciles of distribution in the population, due to lack of brand specificity in the FFQ to accurately estimate absolute intake.

[11]we assigned the highest score to moderate, and the worst score to heavy, alcohol consumers. Nondrinkers received a score of 2.5. We used gender-specific cutoffs, because the health effects of alcohol are seen at lower quantities in women than in men. One drink is 4 oz of wine, 12 oz of beer, or 1.5 oz of liquor (1 oz = 28.35 g).

Supplement table 2. Definition of Healthy Lifestyle behaviors

**Lifestyle behaviors**	**Healthy**	**Unhealthy**
Smoking	Never smoking or past smoking	current smoking
Alcohol intake	never-regular drinkers, weekly drinkers, and moderate daily drinkers (drinking <25g of pure alcohol in men; <15g in women per day)	drinking ≥25g of pure alcohol in men; ≥15g in women per day
Physical activity	Meets any of the following 3 criteria: 3 days of vigorous activity of at least 20min/d; 5 days of moderate intensity activity or walking of >30min/d for >10minutes at a time; achieving at least 600 MET-min/wk.	Does not meet one of the three criteria
Diet quality	Highest 40% AHEI-2010 score	Lower 60% AHEI-2010 score

Abbreviations:AHEI-2010 = Alternative Healthy Eating Index-2010; MET; multiples of resting energy expenditure

Supplement table 3. Univariate analysis for each healthy lifestyle factors and all-cause and CVD mortality

Factors	Statistics	All-cause mortality		CVD mortality	
		HR (95%CI)	P value	HR (95%CI)	P valuee
Smoking status					
Never/former	9354 (80.5)	1.0		1.0	
Current	2268 (19.5)	1.4 (1.2, 1.7)	<0.001	1.3 (1.0, 1.6)	0.043
Alcohol intake					
None/light	10642 (91.6)	1.0		1.0	
Excessive[a]	980 (8.4)	1.1 (0.9, 1.4)	0.285	1.3 (1.0, 1.8)	0.091
Physical activity					
Physically active[b]	9821 (84.5)	1.0		1.0	
Not at all active	1801 (15.5)	1.8 (1.5, 2.1)	<0.001	1.8 (1.4, 2.2)	<0.001
Diet quality					
Highest 40% AHEI-2010[c] score	4664 (40.1)	1.0		1.0	
Lower 60% AHEI-2010 score	6958 (59.9)	1.3 (1.1, 1.5)	0.001	1.5 (1.2, 1.8)	<0.001

[a]Excessive was defined as drinking ≥25g of pure alcohol in men; ≥15g in women per day.

[b]Physically active was defined as 3 days of vigorous activity of at least 20min/d, or 5 days of moderate intensity activity or walking of >30min/d for >10minutes at a time, or achieving at least 600 MET-min/wk.

[c] AHEI-2010: Alternative Healthy Eating Index-2010

Supplement table 4. Risk of incident all-cause and cardiovascular disease mortality according to lifestyle categories (exclude deaths occurred within 1 years of follow-up duration)

Lifestyle categories	Cases/ controls	**Crude Model**		**Model 1**		**Model 2**	
		HR(95%CI)	P value	HR(95%CI)	P value	HR(95%CI)	P value
**All-cause mortality**							
favorable lifestyle	128/2920	Ref.		Ref.		Ref.	
Intermediate lifestyle	351/5417	1.51 (1.24,1.86)	<0.001	1.29 (1.05, 1.55)	0.015	1.28 (1.04, 1.57)	0.020
unfavorable lifestyle	248/2491	2.30 (1.86,2.85)	<0.001	1.48 (1.17, 1.78)	0.001	1.43 (1.14, 1.79)	0.002
P for trend		<0.001		0.001		0.005	
**CVD mortality**							
favorable lifestyle	55/2993	Ref.		Ref.		Ref.	
Intermediate lifestyle	172/5596	1.73 (1.28, 2.34)	<0.001	1.47 (1.08, 1.99)	0.013	1.50 (1.09, 2.04)	0.011
unfavorable lifestyle	115/2624	2.48 (1.80, 3.42)	<0.001	1.65 (1.18, 2.31)	0.003	1.62 (1.15, 2.29)	0.006
P for trend		<0.001		0.004		0.008	

Model I: adjusted for age, gender

Model II: adjusted for age, gender, BMI, waist circumference, hypertension and anti-hypertensive drugs, stroke.

Supplement table 5. Risk of incident all-cause and cardiovascular disease mortality according to lifestyle categories (excluded participants with stroke and myocardial infarction at baseline)

Lifestyle categories	Cases/ controls	**Crude Model**		**Model 1**		**Model 2**	
		HR(95%CI)	P value	HR(95%CI)	P value	HR(95%CI)	P value
**All-cause mortality**							
favorable lifestyle	136/2866	Ref.		Ref.		Ref.	
Intermediate lifestyle	372/5287	1.52 (1.25,1.85)	<0.001	1.29 (1.06, 1.57)	0.012	1.27 (1.04, 1.56)	0.018
unfavorable lifestyle	257/2417	2.26 (1.83,2.78)	<0.001	1.46 (1.18, 1.82)	<0.001	1.39 (1.12, 1.74)	0.003
P for trend		<0.001		<0.001		0.004	
**CVD mortality**							
favorable lifestyle	55/2947	Ref.		Ref.		Ref.	
Intermediate lifestyle	181/5478	1.83 (1.35, 2.47)	<0.001	1.54 (1.14, 2.09)	0.005	1.56 (1.09, 2.13)	0.005
unfavorable lifestyle	115/2559	2.50 (1.81, 3.45)	<0.001	1.66 (1.19, 2.32)	0.003	1.62 (1.15, 2.28)	0.006
P for trend		<0.001		0.004		0.009	

Model I: adjusted for age, gender

Model II: adjusted for age, gender, BMI, waist circumference, hypertension, and anti-hypertensive drugs.

A

Subgroup	Case, n (%) favorable (ref.)	intermediate	unfavorable	HR (95%CI) intermediate vs. ref	unfavorable vs. ref	P for interaction
Age, years						0.896
<65	31 (1.5%)	69 (1.9%)	57 (3.5%)	1.18 (0.77,1.81)	1.34 (0.83,2.16)	
≥65	97 (10.2%)	282 (13.3%)	191 (17.5%)	1.29 (1.02,1.63)	1.41 (1.09,1.83)	
Gender						0.423
Men	54 (7.8%)	75 (9.4%)	195 (9.4%)	1.31 (0.96,1.78)	1.33 (0.98,1.82)	
Women	74 (3.1%)	176 (4.5%)	53 (7.9%)	1.22 (0.92,1.60)	1.59 (1.11,2.29)	
Hypertension						0.775
Yes	70 (7.0%)	181 (9.2%)	123 (12.2%)	1.19 (0.90,1.58)	1.33 (0.97,1.82)	
No	58 (2.8%)	170 (4.5%)	125 (7.2%)	1.38 (1.02,1.87)	1.54 (1.10,2.14)	
Stroke						0.892
Yes	3 (7.0%)	10 (10.1%)	9 (13.4%)	1.03 (0.27,3.98)	1.04 (0.25,4.42)	
No	124 (4.1%)	340 (6.0%)	237 (8.9%)	1.28 (1.04,1.58)	1.44 (1.14,1.81)	
WC, cm						0.171
<90 in men or <85 in women	109 (4.6%)	280 (6.4%)	210 (9.8%)	1.06 (0.84,1.34)	1.10 (0.85,1.41)	
≥90 in men or ≥85 in women	19 (2.7%)	71 (5.0%)	38 (6.3%)	1.34 (0.70,2.54)	1.97 (0.98,3.97)	
BMI, kg/m2						0.006
<24	100 (5.4%)	252 (7.1%)	183 (10.2%)	1.10 (0.87,1.39)	1.16 (0.90,1.50)	
≥24	25 (2.1%)	93 (4.3%)	60 (6.6%)	1.98 (1.27,3.09)	2.70 (1.66,4.39)	

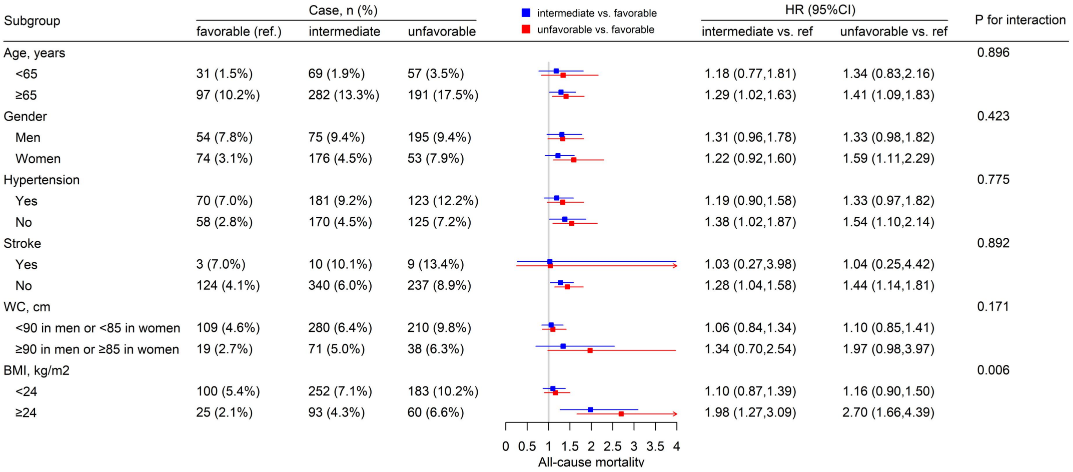

B

Subgroup	Case, n (%) favorable (ref.)	intermediate	unfavorable	HR (95%CI) intermediate vs. ref	unfavorable vs. ref	P for interaction
Age, years						0.985
<65	10 (0.5%)	30 (0.8%)	24 (1.5%)	1.61 (0.78,3.31)	1.70 (0.77,3.78)	
≥65	45 (4.7%)	142 (6.7%)	91 (8.3%)	1.45 (1.03,2.05)	1.55 (1.06,2.27)	
Gender						0.318
Men	19 (2.8%)	84 (4.5%)	88 (4.3%)	1.82 (1.09,3.03)	1.69 (1.01,2.81)	
Women	36 (1.5%)	88 (2.3%)	27 (4.0%)	1.27 (0.85,1.90)	1.69 (1.02,2.82)	
Hypertension						0.668
Yes	34 (3.4%)	96 (4.9%)	62 (6.2%)	1.77 (1.08,2.90)	1.91 (1.11,3.29)	
No	21 (1.0%)	76 (2.0%)	53 (3.1%)	1.34 (0.89,2.00)	1.45 (0.93,2.26)	
Stroke						0.621
Yes	3 (7.0%)	7 (7.1%)	8 (11.9%)	1.54 (1.12,2.12)	1.66 (1.16,2.36)	
No	51 (1.7%)	165 (2.9%)	107 (4.0%)	0.69 (0.17,2.87)	0.91 (0.20,4.09)	
WC, cm						0.048
<90 in men or <85 in women	48 (2.0%)	143 (3.3%)	94 (4.4%)	1.38 (0.97,1.98)	1.25 (0.85,1.84)	
≥90 in men or ≥85 in women	7 (1.0%)	29 (2.0%)	21 (3.5%)	1.44 (0.52,4.02)	2.88 (1.00,8.28)	
BMI, kg/m2						0.056
<24	43 (2.3%)	130 (3.7%)	85 (4.7%)	1.32 (0.93,1.88)	1.28 (0.87,1.89)	
≥24	10 (0.9%)	40 (1.8%)	27 (3.0%)	2.16 (1.08,4.33)	3.30 (1.55,7.01)	

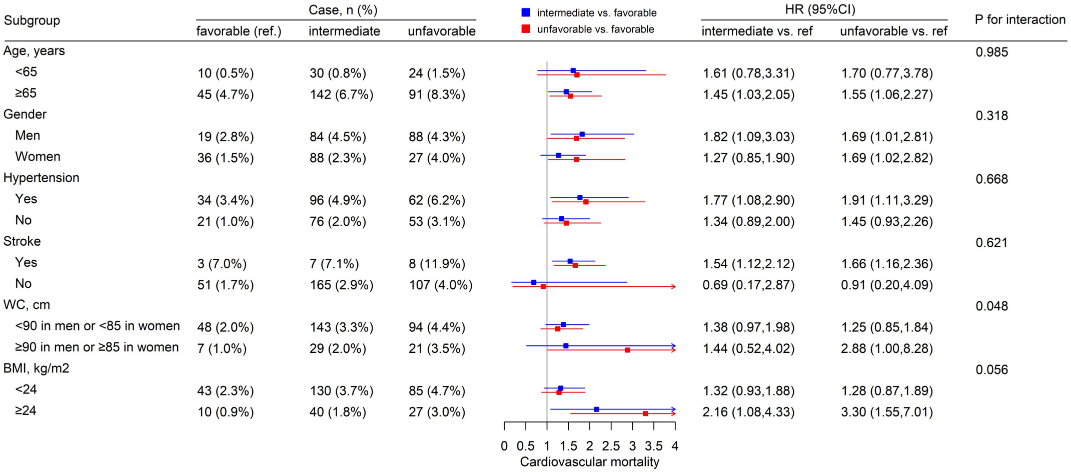

Supplement figure 1. The subgroup analysis for the lifestyle on all-cause (A) and CVD (B) mortality (excluded deaths occurred within 1 years of follow-up duration)
